# Chronic Citalopram Administration Causes a Sustained Suppression of Serotonin Synthesis in the Mouse Forebrain

**DOI:** 10.1371/journal.pone.0006797

**Published:** 2009-08-27

**Authors:** Gerard Honig, Minke E. Jongsma, Marieke C. G. van der Hart, Laurence H. Tecott

**Affiliations:** 1 Neuroscience Graduate Program, University of California San Francisco, San Francisco, California, United States of America; 2 Department of Psychiatry and Center for Neurobiology and Psychiatry, University of California San Francisco, San Francisco, California, United States of America; 3 Brains On-Line, San Francisco, California, United States of America; Pennsylvania State University, United States of America

## Abstract

**Background:**

Serotonin (5-HT) is a neurotransmitter with important roles in the regulation of neurobehavioral processes, particularly those regulating affect in humans. Drugs that potentiate serotonergic neurotransmission by selectively inhibiting the reuptake of serotonin (SSRIs) are widely used for the treatment of psychiatric disorders. Although the regulation of serotonin synthesis may be an factor in SSRI efficacy, the effect of chronic SSRI administration on 5-HT synthesis is not well understood. Here, we describe effects of chronic administration of the SSRI citalopram (CIT) on 5-HT synthesis and content in the mouse forebrain.

**Methodology/Principal Findings:**

Citalopram was administered continuously to adult male C57BL/6J mice via osmotic minipump for 2 days, 14 days or 28 days. Plasma citalopram levels were found to be within the clinical range. 5-HT synthesis was assessed using the decarboxylase inhibition method. Citalopram administration caused a suppression of 5-HT synthesis at all time points. CIT treatment also caused a reduction in forebrain 5-HIAA content. Following chronic CIT treatment, forebrain 5-HT stores were more sensitive to the depleting effects of acute decarboxylase inhibition.

**Conclusions/Significance:**

Taken together, these results demonstrate that chronic citalopram administration causes a sustained suppression of serotonin synthesis in the mouse forebrain. Furthermore, our results indicate that chronic 5-HT reuptake inhibition renders 5-HT brain stores more sensitive to alterations in serotonin synthesis. These results suggest that the regulation of 5-HT synthesis warrants consideration in efforts to develop novel antidepressant strategies.

## Introduction

Depression is a devastating illness and one of the major causes of disability in the world, affecting over 120 million people [1], [2]. Selective serotonin reuptake inhibitors (SSRIs) are widely prescribed as a first-line treatment for depression and many other psychiatric disorders [3]–[5]. The primary pharmacological activity of SSRIs is inhibition of the serotonin transporter (SERT) (P31645.1, *UniProtKB/Swiss-Prot*), which is responsible for the reuptake of serotonin (5-HT) from the extracellular space back into the nerve terminals that release it [6], [7]. Inhibition of this transport alters the spatiotemporal dynamics of serotonin signaling such that activity in the serotonergic neuron causes greater and more prolonged increases in extracellular serotonin than would normally occur [8]–[12].

SSRIs are generally administered continuously for months or years, often indefinitely; however, the precise effects of chronic reuptake inhibition on serotonin and serotonergic neurotransmission are not completely understood [13]–[20]. Although the primary pharmacological targets of SSRIs have been well characterized for decades, several issues regarding their use remain unresolved. Clinical evidence suggests that while SSRIs are generally effective for the treatment of many psychiatric disorders, a substantial number of patients will not respond to the SSRI initially prescribed or even to any SSRI, and other patients will attain only a partial remission of symptoms [5], [21], [22]. Another possible limitation of SSRI treatment is a latency of several weeks for therapeutic effects to occur [6], [14], [23], [24] (but see [25]). The mechanisms downstream of SERT blockade that are responsible for the therapeutic effects of SSRIs remain unknown, despite recent advances [6]. Given the high affinity of SSRIs for SERT [26], it is generally believed that these downstream events are initiated and sustained by effects on serotonergic neurotransmission [27]. Altogether, efforts to develop novel antidepressant strategies are hampered by a lack of fundamental understanding of how SSRIs affect multiple aspects of brain function.

SSRIs affect, secondarily to reuptake inhibition, many aspects of serotonergic neurotransmission, including autoreceptor function and serotonergic neuron activity [14], [28]. Some of these effects have been proposed to influence therapeutic response [14], [29]. In particular, 5-HT synthesis warrants consideration as a factor in SSRI efficacy. In the brain, serotonin is synthesized from the dietary precursor tryptophan through the action of the tryptophan hydroxylase enzyme (TPH2; accession *Q8IWU9,* UniProtKB/Swiss-Prot), which is expressed, in the brain, exclusively in serotonergic neurons. In vitro, the rate of neurotransmitter synthesis is an important factor in monoaminergic physiology [30]. In vivo, 5-HT synthesis rate is regulated by many factors, such as stress [31]–[33] and the availability of tryptophan [34]–[36]. Pharmacological inhibition of 5-HT synthesis can induce a rapid relapse of depression symptoms in SSRI-treated patients with remitted depression, an effect which is not readily observed in subjects with no history of SSRI administration [27], [37]–[44] (also see [45]). Tryptophan itself is considered to be ineffective as an antidepressant [46]; however, tryptophan co-administered with a 5-HT reuptake inhibitor may be more effective as an antidepressant than the reuptake inhibitor administered alone [47]–[50].

These findings raise the question of how chronic SSRI administration itself might affect 5-HT synthesis. There is substantial evidence that acute administration of SSRIs suppresses serotonin synthesis throughout the brain [51]–[57]. SSRI administration can rapidly trigger physiological responses, such as suppression of serotonergic neuronal activity, which gradually dissipate upon chronic treatment [14], [28], [58]. These adaptations may be required for the beneficial effects of antidepressants to emerge [14], [24]. In patients, SSRIs are generally administered continuously for months or years. It is not clear how chronic, continuous administraton of SSRIs affects 5-HT synthesis rate. Prior studies addressing this issue have reported contradictory results, perhaps due to methodological issues [55], [59]–[62]. For several of these studies [59]–[61], the SSRI was administered by repeated injection, which can lead to large daily fluctuations in plasma drug levels [63]. In addition, 5-HT synthesis was assessed some time after the final SSRI injection, to allow the drug to ‘wash out’ of circulation. Drug washout may induce physiological changes which are opposite to the effect of the drug continuously administered [64], [65]. For the other studies addressing this issue, 5-HT synthesis was estimated using an assay whose validity is not universally accepted [55], [62], [66]–[68]. (For a review of these studies, see Discussion.)

Although SSRI treatment produces robust increases in extracellular 5-HT, there is evidence that SSRI administration can actually deplete brain stores of 5-HT and of its major metabolite, 5-hydroxyindoleacetic acid (5-HIAA) [64], [69]–[77], as would be predicted if 5-HT synthesis were suppressed and serotonergic neurons were unable to effectively recapture released 5-HT.

In this study, we explored how chronic reuptake blockade affects forebrain 5-HT synthesis rate and forebrain 5-HT and 5-HIAA content over the course of chronic treatment in mice. We chose to administer citalopram; although many SSRIs such as fluoxetine and paroxetine have significant pharmacological interactions with targets other than SERT [78]–[80], citalopram (CIT) is extremely selective for SERT [26]. To address limitations of previous studies, we used osmotic minipumps, which deliver drug at a constant rate, to generate steady-state blood concentrations of citalopram; we omitted drug washout; and we quantified plasma citalopram levels in order to determine whether they approximated clinically relevant concentrations. In order to address whether CIT administration renders 5-HT stores more sensitive to the suppression of 5-HT synthesis, we also examined forebrain 5-HT and 5-HIAA content in CIT- and vehicle-treated mice following acute inhibition of amino acid decarboxylase.

## Results

**Figure 1:**
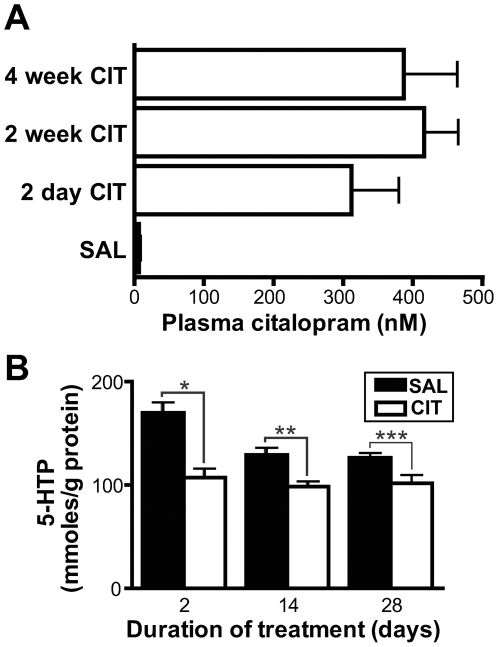
Chronic citalopram treatment: Plasma drug concentrations and inhibitory effect on 5-HT synthesis. A. Plasma levels of CIT were assessed by HPLC-MS in C57BL6/J adult male mice following 2 days, 2 weeks or 4 weeks of citalopram (CIT) (10% solution) or saline (SAL) administration by osmotic minipump (0.25 µL/hour). As no significant amount of citalopram was found in mice implanted with vehicle-filled minipumps for 2, 14 or 28 days, these groups were combined. Numbers of mice: 2-day CIT, n = 5; 2-week CIT, n = 10; 4-week CIT, n = 4; SAL, n = 19. B. 5-HTP accumulation, an index of 5-HT synthesis, in the forebrains of mice following chronic administration of CIT or vehicle by osmotic minipump. NSD-1015 (100 mg/kg IP) was injected 30 minutes before sacrifice. 5-HTP accumulation was significantly suppressed at all time points (*p<0.001, **p<0.01, ***p<0.05, Bonferroni post-ANOVA test). Numbers of mice: 2-day CIT-treated, n = 6; 2-day SAL-treated, n = 6; 2-week CIT-treated, n = 8; 2-week SAL-treated, n = 7; 4-week CIT-treated, n = 7; 4-week SAL-treated, n = 10.

Male adult C57BL6/J mice were treated using osmotic minipumps to deliver a 10% citalopram or saline vehicle solution at a constant low rate (0.25 µL/hour) for 2, 14 or 28 days. The dose and route of administration were chosen to approximate typical pharmacokinetics in patients, in whom CIT rapidly reaches a steady-state plasma concentration with relatively little variation throughout the day [81]–[83]. We used high performance liquid chromatography coupled with mass spectroscopy (HPLC-MS) to quantify CIT concentrations in plasma collected at the time of sacrifice. Plasma levels in CIT-treated mice ranged from 176 to 661 nM, with a mean of 383 nM (Fig. 1A). These levels are comparable to steady-state CIT concentrations found in the blood of patients receiving a typical dose of citalopram. Fredricson Overo et al [84] and Pederson et al [85] observed a mean CIT concentration of approximately 245 nM in patients receiving 40 mg per day, whereas Dufour et al observed blood levels between 157 and 616 nM with 40–60 mg/day dosage [86]. In CIT-treated mice, plasma levels were not significantly affected by treatment duration (one-way ANOVA test for effect of treatment duration, p = 0.48).

5-HT synthesis rate was assessed using the decarboxylase inhibition method. 3-hydroxybenzylhydrazine dihydrochloride (NSD-1015) was injected intraperitoneally (IP) 30 minutes prior to sacrifice (100 mg/kg). Under normal circumstances, the amino acid decarboxylase enzyme (AADC, accession *P20711,* UniProtKB/Swiss-Prot) catalyzes the decarboxylation into 5-HT of 5-hydroxytryptophan (5-HTP), the product of tryptophan hydroxylation. NSD-1015 inhibits AADC, leading to the accumulation of 5-hydroxytryptophan (5-HTP), which is normally present only in minute concentrations due to its rapid decarboxylation. 5-HTP can be precisely identified and quantified by HPLC coupled to electrochemical detection (HPLC-ED) [35], [56], [87]–[90]. Since tryptophan hydroxylation is the rate-limiting reaction in 5-HT synthesis, the amount of 5-HTP that accumulates over a specific period of time is considered an index of the rate of 5-HT synthesis [35], [51], [53], [56], [87]–[91]. The dose of NSD-1015 that we chose is the lowest dose which produces near-maximal inhibition of amino acid decarboxylation in mice [87]. We did not observe any fatalities with this dose of NSD-1015.

**Figure 2:**
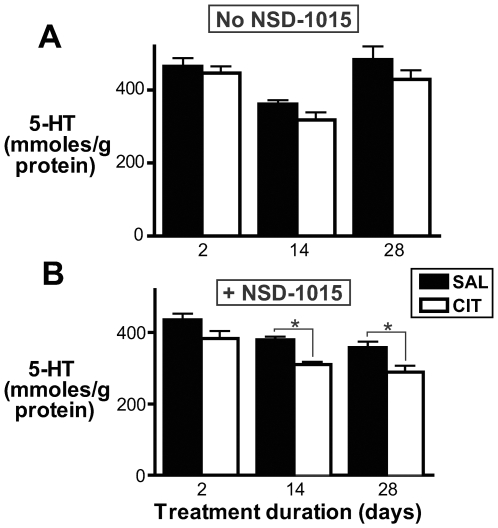
Effect of chronic citalopram treatment on forebrain 5-HT content. A. 5-HT forebrain content in mice treated chronically with CIT or vehicle. 5-HT content was not significantly affected by CIT treatment at any single time point (p>0.05, Bonferroni post-ANOVA test). However, CIT treatment caused a significant overall reduction in 5-HT as determined by ANOVA (p<0.05). Numbers of mice: 2-day CIT-treated, n = 8; 2-day SAL-treated, n = 8; 2-week CIT-treated, n = 8; 2-week SAL-treated, n = 9; 4-week CIT-treated, n = 7; 4-week SAL-treated, n = 7. B. 5-HT forebrain content in mice treated chronically with CIT or vehicle and acutely with NSD-1015 (100 mg/kg IP) 30 minutes before sacrifice. Forebrain 5-HT content was reduced in mice treated with CIT for 2 weeks (*p<0.01, Bonferroni post test) and 4 weeks (*p<0.01) but not 2 days (p>0.05). Numbers of mice: 2-day CIT-treated, n = 6; 2-day SAL-treated, n = 6; 2-week CIT-treated, n = 8; 2-week SAL-treated, n = 7; 4-week CIT-treated, n = 7; 4-week SAL-treated, n = 10.

**Figure 3:**
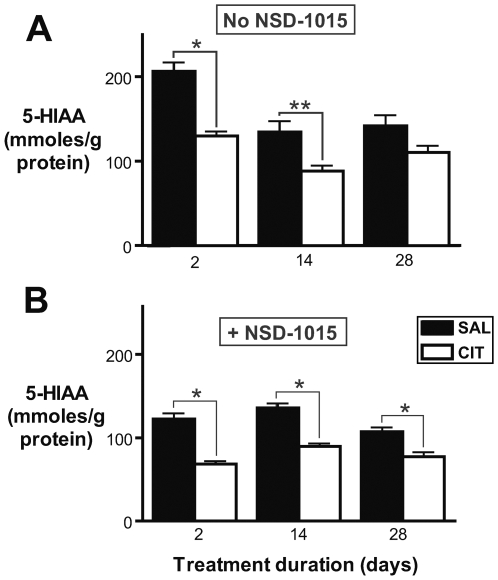
Reduced forebrain 5-HIAA in mice treated with CIT. A. 5-HIAA forebrain content in mice treated chronically with CIT or vehicle. 5-HIAA content was significantly lower in mice treated with CIT for 2 days (*p<0.001, Bonferroni post-ANOVA test) or 2 weeks (**p<0.01) but not 4 weeks (p>0.05). Numbers of mice: 2-day CIT-treated, n = 8; 2-day SAL-treated, n = 8; 2-week CIT-treated, n = 8; 2-week SAL-treated, n = 9; 4-week CIT-treated, n = 7; 4-week SAL-treated, n = 7. B. 5-HIAA forebrain content in mice treated chronically with CIT or vehicle and acutely with NSD-1015 (100 mg/kg IP) 30 minutes before sacrifice. 5-HIAA content was lower in CIT-treated mice at all time points (*p<0.001, Bonferroni post-ANOVA test). Numbers of mice: 2-day CIT-treated, n = 6; 2-day SAL-treated, n = 6; 2-week CIT-treated, n = 8; 2-week SAL-treated, n = 7; 4-week CIT-treated, n = 7; 4-week SAL-treated, n = 10.

For each time point, CIT- and vehicle-treated mice and samples were handled in parallel by an experimenter blinded to treatment group. To determine whether the duration of minipump implantation had an effect on forebrain 5-HTP accumulation in vehicle-treated mice, we performed a one-way ANOVA analysis of the effect of treatment duration on 5-HTP accumulation in SAL-treated mice. In order to evaluate differences between pairs of groups while adjusting for multiple comparisons, we also performed Bonferroni post tests. We observed a significant effect of treatment duration on 5-HTP accumulation in vehicle-treated mice (p = 0.0004, ANOVA) (Fig. 1B). Post tests confirmed that there was significant difference in 5-HTP accumulation between 2-day and 2-week SAL-treated groups (p<0.01) and between 2-day and 4-week SAL-treated groups (p<0.001) but not between 2-week and 4-week SAL-treated groups (p>0.05). We performed identical ANOVA analyses to test for the effect of treatment duration on other neurochemical parameters in SAL-treatment mice. Treatment duration had a significant effect on forebrain 5-HT and 5-HIAA in mice treated with NSD-1015 (p = 0.009 and p = 0.005, respectively) and not treated with NSD-1015 (p = 0.002 and p = 0.0005) (Figs. 2 and 3).

Citalopram treatment resulted in a sustained suppression of 5-HT synthesis rate in the forebrain (Fig. 1B). We performed a two-way ANOVA analysis to test for the effects of drug administration and treatment duration on 5-HT synthesis in mice treated with SAL or CIT for 2, 14 or 28 days. We also performed a Bonferroni post test to evaluate the effect of treatment at each individual time point. CIT treatment had a significant overall effect on 5-HT synthesis (p<0.0001, ANOVA). Treatment duration also had a significant effect and there was an interaction between treatment and treatment duration (p = 0.0015 and p = 0.026, respectively, ANOVA). 5-HTP accumulation was significantly lower in CIT-treated mice than in control mice at all time points. The effect of CIT appeared to be greatest following 2 days of treatment (36% decrease in mean 5-HTP accumulation, p<0.001, Bonferroni post test) and was comparable at 2 weeks (24% decrease, p<0.01) and 4 weeks (20% decrease, p<0.05). These results indicate that 5-HT synthesis remains significantly inhibited following a prolonged treatment with a highly selective SSRI at a clinically relevant dose.

To determine whether SSRI-induced reduction in 5-HT synthesis would lead to decreased brain 5-HT content, we examined 5-HT content in the forebrains of mice treated for 2, 14 and 28 days with CIT or vehicle (Fig. 2A). For this experiment, no NSD-1015 was administered prior to sacrifice. Data were analyzed as for 5-HTP (above). A significant overall effect of treatment on 5-HT was observed (p = 0.04, ANOVA); however, Bonferroni post tests did not demonstrate a significant effect of CIT treatment at any single time point (p>0.05, all time points). These data therefore do not allow us to draw an unequivocal conclusion; however, they suggest that CIT treatment with a clinically relevant dose may have the potential to cause a modest reduction in forebrain 5-HT content.

Multiple mechanisms may exist to buffer brain 5-HT content and stabilize neurotransmission in the face of reduced 5-HT synthesis. One potential mechanism for buffering brain 5-HT content is reduced intracellular degradation of 5-HT into 5-HIAA. To test whether SSRI administration affects 5-HT degradation, we quantified brain 5-HIAA content using HPLC-ED (Fig. 3A & B). Brain 5-HIAA content was reduced in CIT-treated mice, both with and without NSD-1015 administration (p<0.0001, ANOVA) (Fig. 3). In the absence of NSD-1015 treatment, the magnitude of the CIT effect was comparable after 2 days (36% decrease, p<0.001, Bonferroni post test) and 2 weeks of treatment (39% decrease, p<0.01) and was not significant at 28 days of treatment (p>0.05). The ratio of 5-HT to 5-HIAA has been considered to be an index of 5-HT turnover. We observed a significant effect of CIT administration on this ratio (p<0.0001, ANOVA), with a significant reduction following 2 days (p<0.001, Bonferroni post test) and 2 weeks (p = 0.01) but not 4 weeks (p>0.05) of treatment (Fig. S1).

To test the hypothesis that 5-HT reuptake inhibition renders brain 5-HT stores more sensitive to the suppression of 5-HT synthesis, we examined forebrain 5-HT content in CIT- and vehicle-treated mice following acute injection of NSD-1015 (100 mg/kg IP 30 minutes before sacrifice). This NSD-1015 treatment is thought to cause a near-complete inhibition of monoamine synthesis in mice [87]. In this condition, chronic CIT administration caused a significant reduction in brain 5-HT content (p<0.0001, ANOVA) (Fig. 2B). This effect was comparable after 2 weeks (18% decrease; p<0.01, Bonferroni post test) and after 4 weeks of treatment (20% decrease; p<0.01) and was not significant after 2 days (p>0.05).

## Discussion

In this study, we demonstrate that chronic treatment with citalopram, a widely prescribed and highly selective SERT inhibitor [3], [26], causes a suppression of 5-HT synthesis in the mouse brain. This effect is most pronounced following 2 days of CIT administration and persists with prolonged treatment. This effect was observed using a clinically relevant dosing regimen; osmotic minipumps were used to deliver CIT at a constant rate, producing stable plasma concentrations in the clinical range [81]–[84]. This effect was observed using a dissection of the entire right hemi-forebrain, suggesting that this effect is occurring in many, perhaps all, regions of the forebrain. Nevertheless, we cannot infer, based on our results, what effect CIT may have on 5-HT synthesis in specific forebrain nuclei or in brain regions not analyzed, such as the raphe nuclei.

Numerous investigators have reported that acute treatment with SSRIs produces a suppression of 5-HT synthesis [51], [53], [54], [56], [57]. Although several previous studies have addressed the issue of how chronic SSRI administration affects 5-HT synthesis, methodological considerations limit the interpretation of these results. Moret et al [59] and Stenfors et al [60] reported increased 5-HT synthesis rate with chronic treatment with citalopram and fluoxetine, respectively, whereas Esteban et al [61] reported no effect of chronic fluoxetine. For these studies, SSRIs were administered by repeated injection and administration was withheld following the chronic treatment and prior to determination of 5-HT synthesis rate in order to allow the SSRI to ‘wash out’. It is notable, however, that SSRI washout can induce rapid changes in serotonergic physiology which are opposite to the effect of the same drug administered continuously [65], [92]. Notably, Trouvin et al reported reduced brain 5-HT and 5-HIAA content following chronic fluoxetine administration; this effect reversed rapidly with washout [64]. As patients typically take SSRIs for months or years without interruption, the clinically relevant physiological effects of SSRIs occur while the drug is at steady-state levels. Therefore, we did not include a washout period in our study design.

In previous studies of chronic SSRIs and 5-HT synthesis, SSRIs were administered by repeated injection [59]–[61]. This treatment regimen can lead to large daily fluctuations in plasma citalopram in rodents [63] which complicate the interpretation of resulting effects. Yamane et al used osmotic minipumps to administer chronic paroxetine without washout and reported reduced brain trapping of the radiotracer α-[^14^C]-methyl-tryptophan, which is consistent with reduced 5-HT synthesis [55]. However, these authors reported no effect of chronic citalopram in wild-type rats [62]. There is disagreement as to whether or not α-[^14^C]-methyl-tryptophan trapping is a reliable measure of 5-HT synthesis [66]–[68]. The decarboxylase inhibition assay used in the present study has the advantage that 5-HTP can be unambiguously identified and quantified by HPLC-ED; however, as a caveat, 5-HT synthesis is assessed while monoamine systems are significantly disrupted by inhibition of synthesis of multiple active neurotransmitters [93].

In vehicle-treated mice, we also observed a significant effect of time on 5-HT synthesis such that 5-HT synthesis was elevated following 2 days of treatment as compared to following 14 or 28 days. Although the design of our study does not allow us to interpret the specific causes for this effect, we may speculate that peri-operative stress could have affected serotonergic function in the 2-day treatment group. Stress influences serotonergic function, particularly 5-HT synthesis [31], [33]; moreover, interactions between neurochemical responses to stress and to SSRI administration have also been reported [94]. Thus, stress and CIT administration may interact to influence our results regarding 5-HT synthesis and other neurochemical measures. As all mice were of the same age at the time of minipump implantation, aging over the course of the experiment could also have affected our results.

How might our data regarding 5-HT synthesis inhibition be explained in terms of monoamine neurophysiology? As SSRI administration causes a rapid increase in extracellular 5-HT [95], Carlsson and colleagues proposed that high extracellular 5-HT exerts a negative feedback control of 5-HT synthesis in serotonergic neurons [96]. The mechanism whereby this might occur remains unknown. Serotonergic autoreceptors could provide a simple and plausible mechanism; in fact, 5-HT autoreceptor activation can suppress 5-HT synthesis *in vivo* [54], [61], [97]. However, activation of the known 5-HT autoreceptors does not appear to be required for acute suppression of 5-HT synthesis by citalopram [54]. Furthermore, the functional activation of autoreceptors is thought to dissipate over the course of SSRI treatment [14], [29] whereas the effect on synthesis persists. Citalopram might also affect 5-HT synthesis by reducing brain availability of tryptophan; however, this is unlikely as many antidepressants have been reported to increase brain tryptophan by inhibiting metabolism of tryptophan in the liver [98]–[101]. Studies of tryptophan hydroxylase enzyme function in CIT-treated mice could elucidate the as-yet unknown mechanisms underlying the effect we observed.

Our results provide an interesting contrast to previous genetic studies of the relationship between 5-HT synthesis and SERT function [102], [103]. Unlike SSRI treatment, reported null mutations in *SLC24A4*, the gene encoding SERT, result in a complete inactivation of SERT function from embryogenesis through adulthood [102], [104], [105]. Also unlike SSRI treatment, these manipulations have initial effects which are completely SERT-specific. In mice, an engineered null mutation in mice causes an increase in 5-HTP accumulation *in vivo* [103]. In *SLC24A4* null mutant rats generated by forward mutagenesis [102], *in vivo* 5-HTP accumulation has not been reported. In brain tissue from both mice and rats, no increase was observed in the *in vitro* maximal enzymatic activity of tryptophan hydroxylase [102], [103], suggesting that the effect of the mouse mutation on 5-HT synthesis is related to a feature of *in vivo* serotonergic circuitry [103]. The difference in the effects of genetic inactivation and pharmacological blockade of SERT may be related to the magnitude and timing of the physiological effects of these manipulations. Although both manipulations cause a large increase in extracellular 5-HT, null mutations also cause a dramatic depletion of tissue 5-HT [102], [103] and may disrupt serotonergic neuron development, as suggested by the reported decrease in serotonin cell number in null mutant mice [105] (but not rats [106]). The mouse null mutation has behavioral effects which are very different from those SSRI administration [105], as does neonatal treatment with SSRIs [78], [107], [108], which also may impair the development of serotonergic neurons [78], [108]. Null mutations in *SLC24A4* may therefore cause a profound disruption in serotonin physiology and may, as a result, trigger homeostatic adaptations in serotonin synthesis which are not engaged with SSRI treatment.

What might be the functional consequences of chronic suppression of 5-HT synthesis? Neurotransmitter synthesis and its regulation are fundamental features of neurophysiology; however, the functional significance of regulation of 5-HT synthesis [31], [109], [110] is not well understood. Prolonged suppression of 5-HT synthesis by SSRI treatment might deplete 5-HT brain stores, which could limit or otherwise affect SSRI response. We did not observe a clear effect of CIT treatment on forebrain 5-HT content. Trouvin et al reported 20–50% decreases in total 5-HT tissue content in several brain regions following a 21-day treatment with fluoxetine [64], [74]. Dygalo et al [71] and Caccia et al [69], [111] reported comparable results, although this effect was not observed in all brain regions examined. Marsteller also observed reduced tissue 5-HT following chronic treatment with CIT [73]. This effect was, however, not observed in two studies of chronic fluoxetine administration [72], [75]. Taken together, these reports suggest that SSRI treatment may produce a modest reduction in brain 5-HT content, with unknown functional consequences.

Multiple mechanisms may exist to buffer neurotransmitter stores and stabilize neurotransmission in the face of reduced synthesis. For example, glutamatergic synaptic transmission is remarkably resistant to manipulation of glutamate synthesis [112]. Under conditions of reuptake blockade, intracellular 5-HT might be buffered by a suppression of 5-HT degradation. In accord with this hypothesis, we observed a reduction of brain 5-HIAA content with CIT treatment, as has been observed previously [64], [69], [71], [72], [74]–[76], [111] (but see [73], [76], [77]). 5-HT degradation may be suppressed due to a direct SSRI-induced inhibition of monoamine oxidases, as has been reported [75], [76] (but see [113]). Reduced 5-HT reuptake could also shift the cellular compartmentalization of 5-HT, increasing the extracellular concentration at the expense of intracellular stores by effectively trapping 5-HT outside cells. This could in principle lead to a reduction in 5-HIAA production, as the monoamine oxidases, which reside in the mitochondrial membrane, can only metabolize intracellular 5-HT. However, available data suggest that less than 1% of total 5-HT is extracellular under normal conditions [114], [115]. Given that extracellular 5-HT increases only several fold with SSRI administration, this mechanism is unlikely to be sufficient to account for the reduction in 5-HIAA we observed.

Although CIT-induced reductions of forebrain 5-HT synthesis were not accompanied by substantial reductions in forebrain 5-HT stores, we hypothesized that chronic reuptake blockade might render 5-HT stores more vulnerable to decarboxylase inhibition. This hypothesis is supported by the fact that serotonergic neurons have two sources of 5-HT: synthesis from tryptophan and reuptake from the extracellular space. When reuptake is inhibited, brain 5-HT content should be more dependent on 5-HT synthesis and might be depleted more rapidly in response to synthesis inhibition. Accordingly, when we challenged mice by administering NSD-1015 acutely prior to sacrifice, brain 5-HT was reduced in mice treated chronically with CIT. In accord with this finding, SERT null mutant animals have much more pronounced depletion of brain 5-HT content than wild-type animals in response to tryptophan depletion [36] and in response to inhibition of AADC [103]; SERT inactivation also leads to exaggerated neurochemical and behavioral responses to drugs that enhance 5-HT synthesis [116], [117]; and tryptophan depletion has marked effects on extracellular 5-HT in SSRI-treated rats but not in control rats [92], [118]. It is not clear why this depletion effect would be observed with 14 or 28 days but not 2 days of treatment. It is possible that after a relatively brief CIT treatment, serotonergic neurons might have a greater reservoir of intracellular 5-HT, or a heightened tendency to retain intracellular 5-HT due to the reduction in neuronal activity which dissipates with extended treatment.

These results suggest that SSRI administration might cause a form of “serotonergic vulnerability” [119] whereby serotonergic neurotransmission becomes more sensitive to environmental or genetic factors that would inhibit 5-HT synthesis, such as an unbalanced diet [120], [121]. In fact, tryptophan depletion has pronounced depressive effects on patients taking SSRIs [27], [37]–[44]. Conversely, genetic deficits in 5-HT synthesis could limit SSRI efficacy. For example, a putative genetic deficiency in 5-HT synthesis in mice [122]–[124] may be associated with blunted responses to citalopram [56], [125]. Genetic influences on tryptophan hydroxylase function have been proposed to affect SSRI response in humans, although present evidence is not conclusive [126]–[131].

Taken together, these data suggest that pharmacological augmentation of 5-HT synthesis might be beneficial for the treatment of depression if administered in conjunction with a 5-HT reuptake inhibitor. In patients, tryptophan augmentation of 5-HT reuptake therapy is supported by a limited number of clinical trials [47], [48]. These data are, however, not definitive [46], [50] and large follow-up clinical trials have not been reported. In an animal model, behavioral and physiological relevance of the suppression of 5-HT synthesis by SSRIs could be assessed by augmenting 5-HT synthesis in SSRI-treated animals using tryptophan loading [92]. Altogether, our results suggest that the regulation of 5-HT synthesis warrants consideration in efforts to develop novel antidepressant strategies.

## Materials and Methods

### Animals & Surgery

All procedures involving mice were approved by the UCSF Institutional Animal Care and Use Committee. 6-week-old male C57BL/6J mice were obtained from Jackson Laboratories and housed under standard conditions (7 AM to 7 PM light; PicoLab 5053 diet *ad libitum*; individually aerated cages; Specific Pathogen Free facility; 3 mice per cage). 2–3 weeks following shipment, mice underwent surgery between 1 and 4 PM. Mice were anesthetized with 2–4% isoflurane and osmotic minipumps (Alzet) were implanted as per manufacturer's instructions. Minipumps (Model 1002 for 2-day and 2-week treatment groups; Model 2004 for 4-week treatment groups) were filled with sterile 0.9% NaCl vehicle (Hospira) or 10% citalopram hydrobromide (provided by Lundbeck A/S) dissolved in vehicle. Citalopram solution was briefly warmed to 37°C to facilitate dissolution and was sterile filtered. Citalopram-filled pumps produced a dose of approximately 24 mg/kg/day.

### Groups & Treatments

Mice were divided into 12 groups. 6 groups of mice were implanted with CIT-filled minipumps and 6 with SAL-filled minipumps. For 6 groups of mice (CIT- and SAL-treated mice sacrificed following 2, 14 or 28 days of treatment), dissections were performed without additional treatment (other than minipump implantation) prior to sacrifice. For the remaining 6 groups of mice (CIT- and SAL-treated mice sacrificed following 2, 14 or 28 days of treatment), all mice received 2 injections prior to sacrifice: saline 1 hour prior and NSD-1015 (3-hydroxybenzylhydrazine dihydrochloride, Sigma, 54880) (100 mg/kg IP) 30 minutes prior. All treatments, dissections and analyses were performed in parallel for groups corresponding to the same time point (e.g., mice treated for 2 weeks with CIT and injected with NSD-1015 prior to sacrifice were dissected and analyzed in parallel with mice treated with SAL for 2 weeks and injected with NSD-1015 prior to sacrifice). Numbers of mice for NSD-1015-treated groups were as follows: 2-day CIT-treated, n = 6; 2-day SAL-treated, n = 6; 2-week CIT-treated, n = 8; 2-week SAL-treated, n = 7; 4-week CIT-treated, n = 7; 4-week SAL-treated, n = 10.. Numbers of mice for non-NSD-1015-treated groups were as follows: 2-day CIT-treated, n = 8; 2-day SAL-treated, n = 8; 2-week CIT-treated, n = 8; 2-week SAL-treated, n = 9; 4-week CIT-treated, n = 7; 4-week SAL-treated, n = 7.

### Sample collection and preparation

All sample preparation and analysis was performed by an experimenter blinded to treatment group. Following the appropriate treatment period, mice were sacrificed by decapitation under brief isoflurane anesthesia between 1 and 4 PM. Trunk blood was collected in K_2_EDTA tubes (365974, BD) and spun at 1000 g for 10 minutes. Plasma supernatant was stored at −80°C. Brains were rapidly dissected by removing the pineal gland, olfactory bulb and cerebellum and sectioning coronally immediately caudal to the hypothalamus. Forebrains were hemisected saggitally. Brain samples were frozen immediately over powdered dry ice and stored at −80°C. Right forebrain samples were then homogenized using a glass mortar and pestle in 600 µL cold 0.1 M perchloric acid (Sigma) and spun at 16000 g for 15 minutes at 4°C. Supernatant was stored at −80°C and analyzed without further dilution.

### Analysis of plasma citalopram

Plasma citalopram was analyzed by HPLC-MS. Separation was performed on a reverse phase 150×4.6 mm Zorbax Eclipse XDB-C8 column (Agilent Technologies) with a Zorbax Eclipse XDB C8 guard column (Agilent). The mobile phase consisted of 50% water and 50% acetonitrile supplemented with 0.1% formic acid and its flow rate was 0.5 mL/min. A post-column make-up flow of acetonitrile with 0.1 % formic acid was added to assist spray formation. For analysis, each 10 µl plasma sample was thoroughly mixed with 90 µl acetonitrile. After 5 min, samples were spun and 30 µL of the supernatant was injected using an autosampler (Shimadzu SIL-10, Kyoto, Japan). Citalopram was detected using an API4000 mass spectrometer consisting of a turbospray interface (Applied Biosystems). Acquisition was performed in positive ionization mode with ion spray voltage set at 5.5 kV and probe temperature of 400°C. The instrument was operated in multi-reaction-monitoring (MRM) mode for detection of citalopram (precursor 325, product ion 262) and a standard, Lu-10-202 (precursor 341, product ion 278). Quantification was performed by the external standard method using the Analyst 1.4.2 data system (Applied Biosystems). Citalopram peak heights were normalized to peak heights of Lu-10-202 internal standard.

### Analysis of brain 5-HT, 5-HIAA and 5-HTP

5-HT, 5-HIAA and 5-HTP were analyzed by HPLC-ED as follows: Mobile phase consisted of 50 mM sodium acetate, 0.51 mM EDTA, 0.9 mM 1-octanesulfonic acid sodium salt, and 14% methanol (pH 4.4) and was delivered at a flow rate of 1 mL/min using a Shimadzu Prominence pump. Analytes were separated using a reversed phase SupelcoSil LC-18-DB 58993 15cm×4.6mm×3um column (Supelco) heated to 30°C. 50 µL samples were cooled to 12°C in a refrigerated tray and injected using a Gilson 231 autosampler. The external standard method was used for quantification. Dihydroxybenzylamine hydrobromide (Sigma, 858781) was added to each sample and used as an internal standard. Fresh standards (Sigma) were prepared in acetic acid and run at beginning of run. Samples were interspersed with quality control brain sample replicates to monitor sensitivity, chromatography and sample degradation. Analytes were detected using a two-electrode electrochemical cell (model 5011, ESA) the first electrode was set at 50 mV for preoxidation. The second electrode was set 250 mV for quantification. A Coulochem II detector (ESA) was used to control the cell. Peak height was measured using EZChrom Elite software (Scientific Software).

### Protein assays

A BCA microplate assay (Pierce 23225) was used for the determination of protein content in perchloric acid homogenates. Homogenates were allowed to equilibrate at room temperature. 12 µL of each sample was added to 200 µL BCA working reagent, prepared as per the manufacturer's instructions, in quadruplicate in a flat-bottomed 96-well plate (Nunc). BSA standards were prepared in 0.1 M perchloric acid. Plates were sealed with adhesive plate covers (ABI) and well vortexed immediately, then incubated at 37°C for 40 min. Plate covers were removed and absorbance at 562 nm was read on a SpectroMax 190 plate reader (Molecular Devices).

### Statistical analysis

For all peaks, peak height was normalized to the height of the internal standard peak and converted to molarity using the appropriate standard. For single factor comparisons of two groups, two-tailed Student's t-test with Welch's correction was applied. For multiple-factor comparison, multi-factor ANOVA with Bonferroni post-test was applied.

## Supporting Information

Figure S1 Reduced forebrain 5-HIAA / 5-HT ratio in mice treated with CIT. 5-HIAA / 5-HT ratio in the forebrains of mice treated chronically with CIT or vehicle. 5-HIAA / 5-HT ratio content was significantly lower in mice treated with CIT for 2 days (*p<0.001, Bonferroni post-ANOVA test) or 2 weeks (**p<0.01) but not 4 weeks (p>0.05). Numbers of mice : 2-day CIT-treated, n = 8; 2-day SAL-treated, n = 8; 2-week CIT-treated, n = 8; 2-week SAL-treated, n = 9; 4-week CIT-treated, n = 7; 4-week SAL-treated, n = 7.
(0.30 MB EPS)
